# Phylogeny and distribution of *Bradyrhizobium* symbionts nodulating cowpea (*Vigna unguiculata* L. Walp) and their association with the physicochemical properties of acidic African soils

**DOI:** 10.1016/j.syapm.2019.02.004

**Published:** 2019-05

**Authors:** Doris K. Puozaa, Sanjay K. Jaiswal, Felix D. Dakora

**Affiliations:** a Department of Crop Sciences, Tshwane University of Technology, Private Bag X680, Pretoria 0001, South Africa; b Department of Chemistry, Tshwane University of Technology, Private Bag X680, Pretoria 0001, South Africa

**Keywords:** Novel *Bradyrhizobium*, Horizontal gene transfer, Canonical correspondence analysis, *nodD*, Housekeeping genes

## Abstract

In the N_2_-fixing symbiosis, the choice of a symbiotic partner is largely influenced by the host plant, the rhizobial symbiont, as well as soil factors. Understanding the soil environment conducive for the survival and multiplication of root-nodule bacteria is critical for microbial ecology. In this study, we collected cowpea-nodules from acidic soils in Ghana and South Africa, and nodule DNA isolates were characterized using 16S–23S rRNA-RFLP, phylogenetic analysis of housekeeping and symbiotic genes, and bradyrhizobial community structure through canonical correspondence analysis (CCA). The CCA ordination plot results showed that arrow of soil pH was overlapping on CCA2 axis and was the most important to the ordination. The test nodule DNA isolates from Ghana were positively influenced by soil Zn, Na and K while nodule DNA isolates from South Africa were influenced by P. The amplified 16S–23S rRNA region yielded single polymorphic bands of varying lengths (573–1298 bp) that were grouped into 28 ITS types. The constructed ITS-dendrogram placed all the nodule DNA isolates in five major clusters at low cut-off of approx. 0.1 Jaccard’s similarity coefficient. The phylogenetic analysis of 16S rRNA and housekeeping genes (*glnII, gyrB,* and *atpD*) formed distinct *Bradyrhizobium* groups in the phylogenetic trees. It revealed the presence of highly diverse bradyrhizobia (i.e. *Bradyrhizobium vignae*, *Bradyrhizobium elkanii*, *Bradyrhizobium iriomotense*, *Bradyrhizobium pachyrhizi*, and *Bradyrhizobium yuanmingense*) together with novel/unidentified bradyrhizobia in the acidic soils from Ghana and South Africa. Discrepancies noted in the phylogenies of some nodule DNA isolates could be attributed to horizontal gene transfer or recombination.

## Introduction

Cowpea was first domesticated in West Africa and has since spread throughout the tropical and semi-arid regions of the world [10]. As a result, Sub-Saharan Africa is the largest producer of cowpea. The legume is known for its high nutritional value (25% protein, 57% carbohydrate) as well as for its high soil improvement ability. Cowpea can get a large proportion of its N requirements from symbiotic N_2_ fixation by rhizobial bacteroids in root nodules [1], [9], [12], [40]. Studies done across Africa on cowpea have shown significantly high but varying levels of symbiotic dependency on N_2_ fixation for its N nutrition, e.g. 59–93% in South Africa, 99% in Ghana, 70–87% in Mozambique and 66% in Botswana [33], [40], [41]. This N_2_-fixing trait underpins the ability of cowpea and other legumes to thrive under low-nutrient conditions in the soils of Sub-Saharan Africa. In the N_2_-fixing symbiosis, legumes can form root nodules with a diverse group of rhizobia. The choice of a symbiotic partner is largely influenced by both the host–plant, the rhizobial symbiont, as well as soil and other biogeographic factors such as rainfall and location [7], [22], [42], [59]. The effect of host plant on the microbial community and population diversity in the rhizosphere has been documented [19], [42]. Within Africa, Chidebe et al. Mohammed et al. and Pule-meulenberg et al. [9], [32], [40] have found differences in the diversity of rhizobia nodulating different cowpea varieties in different geographic locations.

Although cowpea is generally nodulated by slow-growing rhizobia belonging to the genus *Bradyrhizobium*, fast-growing rhizobia have also been reported to nodulate this species [9], [48]. Earlier phylogentic studies have shown the ability of cowpea to nodulate with different microsymbionts, which included *Ensifer*, *Achromobacter*, *Bradyrhizobium*, *Rhizobium*, and *Microvirga* species [9], [16], [44], [60]. Sequencing the 16S-rRNA gene has become a popular tool for bacterial phylogenic studies. However, it has been reported that this gene is extremely conserved in the *Bradyrhizobium* genus. So, in order to detect higher diversity, the analysis of rapidly evolving genes or regions such as the 16S–23S ITS together with *nif*H gene and/or selected housekeeping genes have been adopted [3], [9], [58]. Also, the phylogenetic analysis of sequences of single locus may inadequately reflect the phylogenetic relationship among bacterial strains. Thus, the use of additional gene loci with great sequence divergence and sufficient conserved sequences for phylogenetic analysis has been recommended as an alternative to the analysis of only the 16S-rRNA gene [11].

The physico-chemical properties of soils play an important role in influencing the diversity of bradyrhizobial communities in specific niches [22], [42]. The association of bradyrhizobial community when combined with environmental measurements do produce enormous amount of noisy data that can be mitigated by multivariate analysis. Multivariate analysis can structure the data in such a way as to separate systematic variation from noise. Canonical correspondence analysis (CCA) is a multivariate method that helps in the estimation and statistical testing of environmental effects on microbial communities [4]. It is often used to determine whether a specific soil environment influences the microbial community.

Since Sub-Saharan Africa is the center of origin of cowpea, it is likely to harbor a wide diversity of rhizobia capable of effectively nodulating cowpea [8], [9], [10]. The choice of Ghana in West Africa and South Africa at the bottom tip of the African continent was to further explore the biogeographic distribution of bacteria nodulating cowpea in its region of origin. Therefore, the aim of this study was to investigate the influence of diverse soil environments on molecular diversity, and the phylogeny of root-nodule bacteria nodulating cowpea genotypes grown at different locations in Ghana as well as in the cowpea-producing region of South Africa in order to understand agricultural and ecological benefits of the legume–rhizobia symbiosis in the two countries.

## Materials and methods

### Description of the research area

Field experiments were conducted in February 2013 in South Africa and during the major cropping season in Ghana (July–November 2013). The field experiment in South Africa was located at Morwe (latitude −25.153, longitude 28.961) in the Nkangala District Municipality of the Mpumalanga Province. In Ghana, the field experiments were situated in the Guinea Savanna of Northern Region and in the Sudan Savanna of Upper East Region. The experiments in the Northern Region were planted at three sites [namely, Savelugu (latitude 9.624722, longitude −0.827778), Gbalahi (latitude 9.433333, longitude −0.766667) and Kpalisogu (latitude 9.405066, longitude −1.002990)], while in the Upper East Region they were planted at Googo (latitude 10.7545041, longitude −0.4879915) and Manga (latitude 11.017331, longitude −0.264352) (Fig. S1). Prior to planting, soil was sampled and analysed for pH (H_2_O), texture, organic matter, organic C [56], total N (using Dry combustion method), P using Bray-2 [5], K, Na, Ca, Cu, Mn, Zn, Fe, Mg, and cation exchange capacity (CEC) (using ammonium acetate method) as described previously [47] (Table S1). During the experimental period, the rainfall was 583.1 mm at the Northern Region site, 488.7 mm in the Upper East Region, and 138.4 mm at Morwe in the Mpumalanga Province.

### Source and type of germplasm used in the study

Seeds of five cowpea genotypes with different seedcoat pigmentations (Black, Cream, Red, Brown and Buff) were obtained from farmers in Northern Ghana, and from breeders at the CSIR-Savannah Agricultural Research Institute (CSIR-SARI), Ghana. They comprised four landraces [Black (Bensogla), Buff (Omondaw), Red (Sazie), and Brown (Bengmnunomnuno)] and one commercial variety [Cream (Apagbaala). These cowpea genotypes have similar growth habits and phenology.

### Seed planting and nodule sampling

The seeds were sown singly or together (Black, Red and Cream) in one hole to see the effect of seed coat colour on rhizobial interaction. The holes were 20 cm within row and 60 cm between rows, and the plots arranged in a randomized complete block design at sampling locations (Fig. S1). At 56 days after sowing during early pod-filling, the roots of 2–3 plants per genotype were carefully excavated for nodule collection. The nodulated roots were separated from stem, placed in well labelled plastic bags, and transported to the laboratory. Soil and debris were gently washed off under tap water, the nodules plucked and placed in vials containing silica gel, and stored at 4 °C prior to DNA isolation.

### Extraction of root nodule bacterial DNA

The stored nodules were rehydrated by soaking in sterilized distilled water, surface-sterilized as described by Somasegaran & Hoben [49] and transferred into 2-ml volume of microcentrifuge tubes. Each single nodule was crushed in a drop of sterilized distilled water using a sterile plastic pestle. The DNA was extracted directly from the nodules using GenElute bacterial genomic DNA extraction kit (Sigma-Aldrich, USA). The DNA purity and concentration was checked using 0.8% agarose gel stained with ethidium bromide in horizontal gel electrophoresis. The extracted nodule bacterial DNA samples were stored at −20 °C for later use.

### PCR-amplification and restriction fragment length polymorphism (RFLP) of the inter-transcribed spacer (ITS) of 16S–23S rRNA region

The 16S–23S rRNA inter-transcribed spacer (ITS) region of the genomic DNA was amplified using rhizobial specific respective primer pairs (Table S2). The ITS region was amplified by using a Thermal Cycler (Bio-RAD T100) in a 25 μl reaction mixture containing 40–50 ng DNA template, 5X MyTaq PCR buffer (1X), 10 pM of each of the primer, 0.5U *Taq* polymerase (Bioline, USA) and PCR grade water in 0.2 ml PCR tube. The PCR amplification was done using the temperature profile described in Table S2. The PCR-amplified products were digested with three endonucleases (*Taq*I, *Hind*III and *Msp*I) separately (Thermo Scientific, Lithuania). The amplified polymorphic bands obtained from different nodule DNAs were considered as the ITS types of the bacterial population. The restriction enzyme-digested ITS fragments (ITS-RFLP patterns) were analysed after migration in 3% (w/v) agarose gel at 85 V for 2.5 h. The presence or absence of homologous bands was scored using a binary approach, and a UPGMA based dendrogram constructed with NTSYSpc 2.2 programme [46].

### PCR amplification, sequencing and phylogenetic analyses of 16S rRNA, housekeeping (*atpD*, *gyrB,* and *gln*II) and symbiotic (*nifH* and *nodD*) genes

The PCR amplification of 16S rRNA, together with three housekeeping and two symbiotic gene regions of the test nodule DNA samples, was done as described above for the ITS-PCR amplification. The primers used and the appropriate temperature conditions employed are listed in Table S2. The PCR-amplified products were examined on gel electrophoresis, and purified using Rapid PCR Cleanup enzymes set purification kit (New England Biolabs, USA). The purified samples were sequenced (Macrogen, Netherlands), and the quality of the sequences checked using BioEdit 7.0.0 software [18]. The obtained sequences were screened for chimeric sequences using UCHIME. The NCBI GenBank database was used to identify closely related species with test strains using the BLASTn program. Reference sequences were aligned with sequences of the test DNA samples using MUSCLE [13] for construction of phylogenetic trees by MEGA 6.0 program [52]. The trees were generated by means of the Kimura-2 parameter model [24] and the evolutionary history inferred using maximum-likelihood method. Bootstrap analysis [14] based on 1000 replications was performed in order to check the stability of grouping results in the phylogenetic trees. The generated sequences were submitted to NCBI GenBank to get accession number (Table S3)

### Environmental influences on microsymbiont population

Canonical correspondence analysis (CCA) was performed with the vegan package (version 2.4–2) [39] of R software [43] with soil environmental factors and ITS-RFLP data of nodule DNA isolates to determine which physico-chemical characteristic of the soil was frequently related to the population distribution of the rhizobia. The correlation of the canonical axes with the explanatory matrix was determined using the general permutation test.

## Results

### Root nodules collected and soil characteristics

A total of 76 cowpea root nodules were collected from acidic soils (pH 4.5–5.5) in Ghana and South Africa. The physico-chemical environment of the test soils is indicated in Table S1. The soil data showed that the Googo site had higher organic matter, total N, K, Na, Cu and Mn when compared to the soils from other sites (Table S1).

### PCR-amplification and RFLP analysis of ITS region

PCR amplification of the ITS region of the 76 test nodule DNA samples produced prominent polymorphic single bands from each nodule DNA of varying lengths between 573 bp to 1298 bp. These polymorphic bands grouped the nodule DNA samples into 28 ITS types (I–XXVIII) (Table 1). The PCR-amplified ITS region was separately digested with each of the four-base cutter restriction endonucleases, *Msp*I and *Taq*I, and the six-base cutter *Hind*III. Restriction digestion of the PCR-amplified ITS products with *TaqI* yielded a maximum of seven DNA fingerprinting profiles (A-G), while *Hind*III and *Msp*I resulted in five (A–E) and 11 (A–K) profiles, respectively (Table 1). Cluster analysis of the combined restriction patterns obtained from the three enzymes was carried out to ascertain similarity in the ITS region of the nodule DNA isolates. The analysis revealed 30 unique ITS-RFLP patterns which grouped in five major clusters (Cluster I–V) at a low cut-off point of approx 0.1 Jaccard’s similarity coefficient (Fig. S2). Nodule DNA samples TUTCT4B, TUTCS4B and TUTCG5C stood alone, indicating that they were very divergent from the other 73 samples (Fig. S2). Cluster I comprised 26 nodule DNA isolates, Cluster II contained 18, while Cluster III had 3, Cluster IV had 11, and Cluster V had 15 nodule DNA isolates (Fig. S2).

**Table 1:** Restriction patterns and typing of ITS (16S–23S rDNA) region of cowpea nodule DNA isolates.

Location	Seedcoat colour	Isolate designation^a^	*TaqI*	*MspI*	*HindIII*	Amplified band length (bp)	ITS type	Combined ITS RFLP pattern
Googo	Black	TUTCG1A	A	A	A	952	XIX	1
	Black	TUTCG1C	G	B	B	1036	XXV	20
	Cream	TUTCG2C	A	A	A	968	XX	1
	Buff	TUTCG3A	A	A	A	952	XIX	1
	Buff	TUTCG3C	A	A	A	952	XIX	1
	Red	TUTCG4A	A	A	A	952	XIX	1
	Red	TUTCG4C	A	A	A	952	XIX	1
	Brown	TUTCG5A	B	C	A	891	XIII	5
	Brown	TUTCG5B	B	D	B	1018	XXIV	27
	Brown	TUTCG5C	B	E	C	1018	XXIV	30
	Black	TUTCG1.1	B	D	B	1000	XXIII	27
	Cream	TUTCG2.1	B	C	A	852	VII	5
	Red	TUTCG4.1	B	C	A	871	X	5

Gbalahi	Black	TUTCT1A	C	F	A	933	XVIII	12
	Black	TUTCTIC	C	G	D	852	VII	18
	Cream	TUTCT2A	C	I	A	852	VII	13
	Cream	TUTCT2B	D	C	A	886	XII	6
	Buff	TUTCT3A	D	C	A	886	XII	6
	Buff	TUTCT3B	D	C	A	886	XII	6
	Buff	TUTCT3C	D	C	A	886	XII	6
	Red	TUTCT4A	C	H	E	933	XVIII	15
	Red	TUTCT4B	–	G	A	871	X	9
	Red	TUTCT4C	D	C	A	871	X	6
	Brown	TUTCT5B	D	C	A	871	X	6
	Brown	TUTCT5C	C	I	A	917	XVI	14
	Cream	TUTCT1.I	C	I	A	933	XVIII	14
	Red	TUTCT4.I	C	C	E	886	XII	16

Kpalisogu	Black	TUTCK1A	–	C	A	886	XII	5
	Black	TUTCK1B	D	C	A	886	XII	6
	Cream	TUTCK2B	D	C	A	871	X	6
	Buff	TUTCK3A	D	C	A	838	VI	6
	Buff	TUTCK3B	F	C	A	852	VII	7
	Red	TUTCK4A	F	C	A	865	IX	7
	Red	TUTCK4C	D	C	A	865	IX	6
	Brown	TUTCK5B	C	H	C	968	XX	17
	Brown	TUTCK5C	C	I	A	968	XX	14
	Cream	TUTCK2.I	A	A	A	952	XIX	1

Morwe	Black	TUTCSA1A	A	C	A	891	XIII	4
	Black	TUTCSA1B	E	A	B	952	XIX	11
	Black	TUTCSA1C	C	J	B	871	X	21
	Cream	TUTCSA2A	A	J	B	859	VIII	22
	Cream	TUTCSA2B	E	A	A	803	IV	11
	Cream	TUTCSA2C	A	C	B	871	X	23
	Buff	TUTCSA3A	A	C	A	763	II	4
	Buff	TUTCSA3B	A	J	A	763	II	2
	Buff	TUTCSA3C	E	A	A	827	V	11
	Red	TUTCSA4A	C	D	B	903	XV	28
	Red	TUTCSA4B	A	C	A	789	III	4
	Red	TUTCSA4C	C	C	A	803	IV	8
	Brown	TUTCSA5A	-	I	B	917	XVI	26
	Cream	TUTCSA2.I	F	C	B	1123	XXVI	29
	Red	TUTCSA4.I	A	C	B	1123	XXIII	23

Manga	Black	TUTCM1C	F	C	A	903	XV	7
	Cream	TUTCM2C	A	C	A	952	XIX	4
	Buff	TUTCM3B	A	-	B	1298	XXVIII	23
	Red	TUTCM4A	A	A	A	1123	XXVI	1
	Red	TUTCM4C	A	C	A	1190	XXVII	4
	Brown	TUTCM5A	A	C	A	922	XVII	4
	Brown	TUTCM5B	C	D	D	573	I	19
	Brown	TUTCM5C	A	C	A	897	XIV	4
	Cream	TUTCM2.I	A	D	B	973	XXI	24
	Red	TUTCM4.I	A	D	B	1000	XXI	24

Savelugu	Black	TUTCS1A	A	D	B	987	XXII	24
	Black	TUTCS1B	A	C	A	838	VI	4
	Black	TUTCS1C	C	F	A	897	XIV	12
	Cream	TUTCS2A	A	C	A	838	VI	4
	Cream	TUTCS2B	A	C	A	827	V	4
	Cream	TUTCS2C	A	K	A	922	XVII	3
	Buff	TUTCS3A	A	C	A	859	VIII	4
	Buff	TUTCS3B	A	C	A	886	XII	4
	Buff	TUTCS3C	A	C	A	878	XI	4
	Red	TUTCS4A	A	A	A	903	XV	1
	Red	TUTCS4B	A	C	F	852	VII	10
	Red	TUTCS4C	A	C	A	838	VI	4
	Black	TUTCS1.I	A	–	A	852	VII	1
	Cream	TUTCS2.I	A	A	B	903	XV	25

a TUT = institute name (Tshwane University of Technology); C = cowpea; G = Googo, T = Tamale (Gbalahi), K = Kpalisogu, SA = South Africa (Morwe), M = Manga, S = Savelugu; 1 = Black, 2 = Cream, 3 = Buff, 4 = Red, 5 = Brown; A–C = to differentiate nodules; .I = intrahole.

Nodule DNA isolated from cowpea plants at Googo contained eight (VII, X, XIII, XIX, XX, XXIII, XXIV, XXV) ITS types (852 bp–1036 bp) which grouped in Clusters I, II and V in the dendrogram. The 14 nodule DNA isolates from Gbalahi revealed five ITS types (852 bp–933 bp) and grouped in Clusters I and II. Also, a group of 10 nodule DNA samples all isolated from cowpea growing at either Kpalisogu and Manga, revealed six ITS RFLP patterns of 7 (VI, VII, IX, X, XII, XIX, XX) and 9 (I, XIV, XV, XVII, XIX, XXI, XXVI, XXVII, XXVIII) ITS types, respectively, which are indicated in Clusters I, II and IV. Again, 14 nodule DNA isolate samples from Savelugu produced 10 (V, VI, VII, VIII, XI, XII, XIV, XV, XVII, XXII) ITS types and revealed seven RFLP patterns which are indicated in Clusters I and IV. In contrast, 15 nodule DNA samples from Morwe recorded 12 (II, III, IV, V, VIII, X, XIII, XV, XVI, XIX, XXI, and XXIII) ITS types and showed 10 ITS-RFLP patterns which can be found in in Clusters I, II, III and V (Table 1 and Fig. S2).

The nodule DNA samples isolated from the cowpea genotypes planted at five locations in Ghana and one location in South Africa were distributed among all clusters. Interestingly, nodule DNA isolates from cowpea with cream and red seedcoat and planted in single holes at Googo (TUTCG2.I and TUTCG4.I), Maanga (TUTCM2.I and TUTCM4.I), Gbalahi (TUTCT1.I and TUTCT4.I) and Morwe (TUTCSA4.I and TUTCSA2.I) seemed to nodulated by the same microsymbionts, as they showed identical RFLP banding patterns and occupied the same cluster in the generated dendrogram (Table 1; Fig. S2). In contrast, nodule DNA isolate samples isolated from cowpea with black seedcoat and co-planted with cream and red cowpea in same hole seemed to be nodulated by diverse microsymbionts compared to the symbionts from root nodules of cream and red cowpea.

### Canonical correspondence analysis (CCA)

**Fig. 1:**
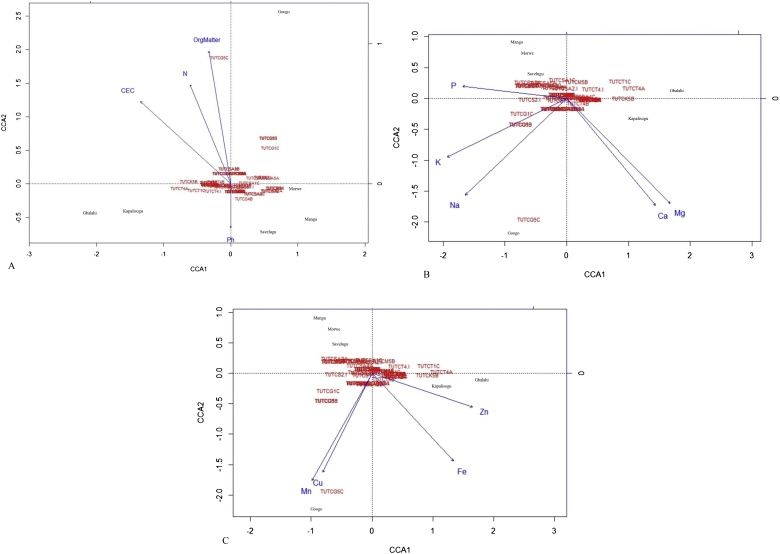
Canonical correspondence analysis (CCA) (A) among chemical soil factors, bradyrhizobial isolates, and sampling locations, (B), among soil macro-elements, bradyrhizobial isolates, and sampling locations and (C) among soil micro-elements, bradyrhizobial isolates, and sampling locations. Permutation tests confirmed the effect of the soil variables as drivers of the rhizobial community (p < 0.05).

CCA is usually used to define the maximum relationship between a community composition and the measured environmental variables that are determinable from ordination biplots of the data. In this study, CCA was applied to understand the correlation of soil environmental factors with bacterial matrixes. Of the soil environmental variables that explain the pattern of bradyrhizobial population profiles across the test locations, the chemical (pH, organic matter, N, CEC), micro- (Zn, Fe, Mn, Cu), and macro-element (Ca, P, Mg, K, and Na) properties of soils were used to correlate with the bradyrhizobial population distribution (Fig. 1A–C; Table 1). Based on these CCA results, the distribution of rhizobial population was affected by the soil environmental variables. In the physico-chemical CCA plot, total mean square contingency coefficient (inertia) was calculated to be 0.147. Inertia was distributed in 0.097 (66.5%) constrained (explainable) and 0.049 (33.5%) unconstrained (unexplainable) (Table S4). With canonical correspondence analysis, and eigen vector analysis, we observed that most of the inertia was carried by the first axis (CCA1). The total inertia (eigen value) in CCA1 was 0.06. The proportion of species variance explained at CCA1 was 41% of total inertia while CCA2, CCA3 and CCA4 explained about 13, 8 and 3.2%, respectively (Table S4). The constructed plot results showed that among all the soil physico-chemical variables tested, soil pH showed stronger correlation with CCA2 as the arrow overlapped on the axis and was the highest importance to the ordination, thus indicating that this variable plays a significant role in shifting or structuring the bradyrhizobial community. Additionally, organic matter formed a smaller angle to the axis, suggesting that organic matter had an effect on the rhizobial community variation along the CCA2 axis (Fig. 1A).

Except for a few, all the nodule DNA isolates were placed in the centre of the ordination plot, suggesting that they coexisted in the community, or were similar in their distribution across the experimental sites.

In the ordination plots, the micro and macro-elements showed a total of 82.9% and 83.9% explainable variations, respectively (Fig. 1B and C). The nodule DNA isolates represented by TUTCT1C, TUTCT4A, TUTCT4.I, TUTCG5B and TUTCG1C from Gbalahi and Googo of Ghana were positively influenced by the presence of the micro-element Zn (*p ≤* 0.05) (Fig. 1B). Bradyrhizobial isolates were positively influenced by P in soil collected from Morwe in South Africa, and by N and K in soil collected from Googo in Ghana (Fig. 1C). Nodule DNA isolate TUTCG5C was highly influenced by soil organic matter (Fig. 1A).

### Phylogenetic analysis of the 16S rRNA gene

Representative nodule DNA isolates were selected from all ITS clusters for sequencing of single band 16S rRNA gene. The generated sequences were subjected to BLAST_n_ analysis using the NCBI database. The results showed high similarity with reference type strains belonging to the *Bradyrhizobium* genus. A 16S rRNA phylogenetic tree built with the sequences obtained split the nodule DNA isolates into four large groups (Groups I–IV) (Fig. S3).

Here, nodule DNA isolates TUTCSA4B, TUTCSA3B and TUTCSA3A (Group IV) obtained from South Africa were most close to *Bradyrhizobium vignae* 7-2^T^ with a sequence identity 98.2–99.4%, while nodule DNA isolates TUTCSA2B (from South Africa) and TUTCS4B, and TUTCT4.I (from Ghana) in Group III were close to *Bradyrhizobium kavangense* 14-3^T^ and *Bradyrhizobium subterraneum* 58 2-1^T^ with sequence identity 99.5%. Nodule DNA isolates TUTCT3A, TUTCG4.I, TUTCG2.I and TUTCT5B (Cluster III) from Ghanaian soil stood as a outgroup. Nodule DNA isolates TUTCSA4C, TUTCSA2A, TUTCSA2.I, TUTCT2A, TUTCT1.I, TUTCSA4A, TUTCSA1C and TUTCT4A (Group I) were very close to *Bradyrhizobium elkanii* USDA 76^T^ and *B. pachyrhizi* PAC48^T^ with 99.2–100% sequence identity. Nodule DNA isolates TUTCG1.I, TUTCG5C, and TUTCT1A, all coming from Ghanaian soils occurred in Group II without any very close *Bradyrhizobium* type reference strain.

### Phylogeny of *atp*D, *gyrB* and *gln*II housekeeping genes

To obtain a clearer resolution of the phylogeny of bradyrhizobia nodulating cowpea in Ghana and South Africa, three highly conserved housekeeping genes (*atp*D, *gyr*B and *gln*II) were sequenced. The amplified band lengths were approximately 550 bp for *atp*D, 700 bp for *gyr*B and 600 bp for *gln*II. The sequence results showed that *atpD* had the highest (62.1%) conserved positions, while variable, parsimony-informative and singleton positions were highest in *gyrB* nucleotide sequences (Table S5).

A phylogenetic tree constructed from *atp*D, *glnII* and *gyrB* gene sequences of the nodule DNA isolates and reference type strains retrieved from NCBI GenBank, placed the nodule DNA isolates into nine different groups (Groups I–IX) (Figs. S4–S6).

The individual housekeeping phylogenetic trees of *atpD, glnII* and *gyrB* showed that the nodule DNA isolates in all nine groups had no close relationship with known reference type *Bradyrhizobium* strains. Except nodule DNA isolates TUTCSA3B, TUTCSA3A, TUTCSA4B, and TUTCSA3C, all the remaining test isolates revealed 93.2–97% sequence identity with the *atpD, glnII* and *gyrB* gene sequences of the reference type *Bradyrhizobium* strains. In the *glnII* and *gyrB* phylogenies, isolates TUTCSA3B, TUTCSA4B and TUTCSA3A grouped with *Bradyrhizobium vignae* 7-2^T^ with 99–100% sequence identity and isolate TUTCSA3C aligned with *Bradyrhzobium arachidis* CCBAU 051107^T^ with 99% identity. With the unavailability of *atpD* sequences of *Bradyrhizobium vignae* 7-2^T^ in NCBI database, isolates TUTCSA3B, TUTCSA4B and TUTCSA3A grouped together without any reference type strains in the phylogeny.

There were discrepancies observed in the phylogenies of three housekeeping genes. Although nodule DNA isolate TUTCT1A stood separately in the *atpD* phylogeny, it also aligned with *B. elkanii* in the *glnII* and *gyrB* phylogenies. Similarly, in the *glnII* phylogeny, isolates TUTCSA2B and TUTCG4.I aligned with *B. elkanii*, but stood alone in the *atpD* and *gyrB* phylogenies in *B. japonicum* group.

### Concatenated sequence and phylogenetic analyses

**Fig. 2:**
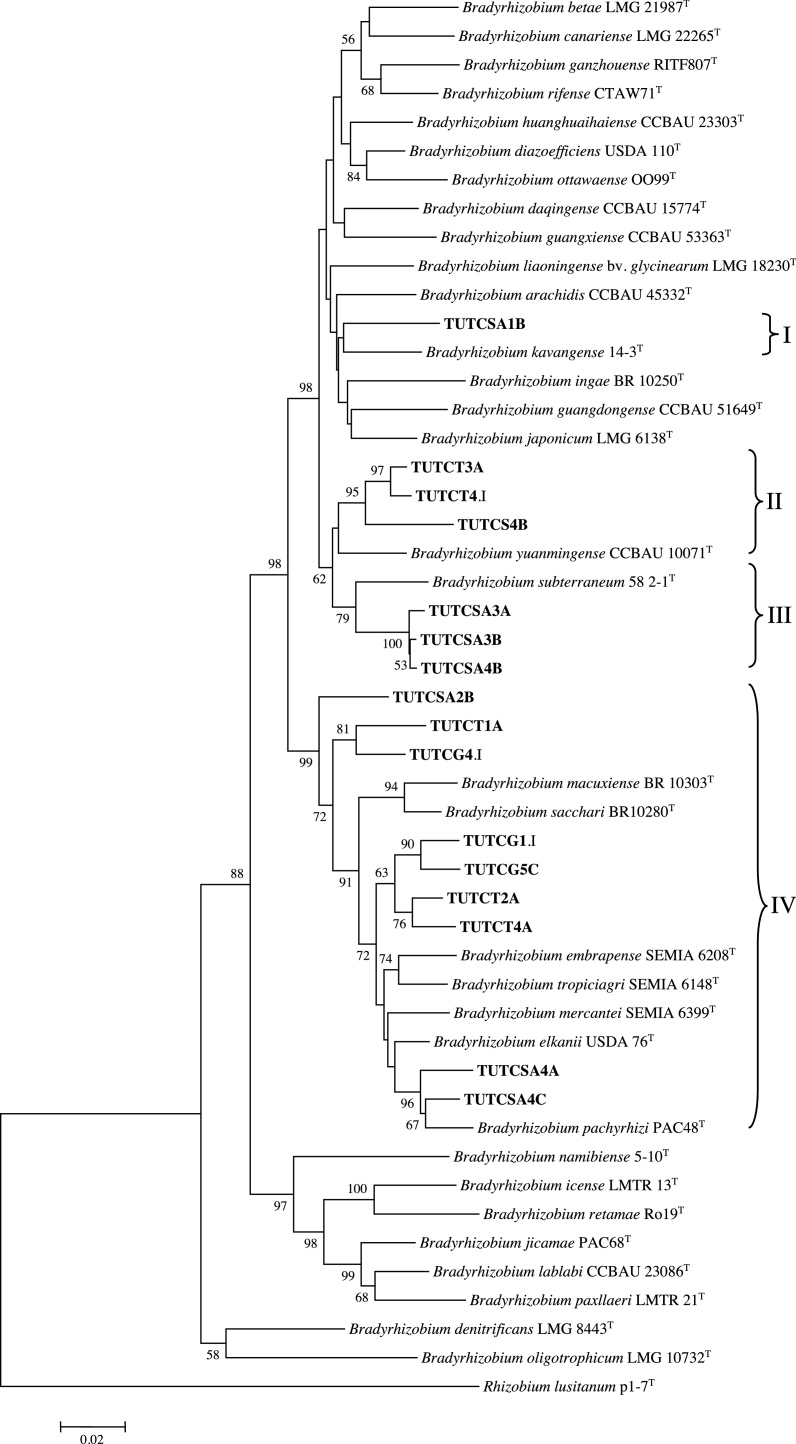
Phylogenetic relationships of concatenate gene (*atpD* + *glnII* + *gyrB*) sequences of cowpea nodule DNA. Phylogeny was inferred using the maximum-likelihood method. The percentages of replicate trees in which the associated taxa clustered together were obtained using bootstrap test with 1000 replications.

To refine the phylogentic positions of the nodule DNA isolates, the concatenated sequences of *atpD* *+* *glnII* *+* *gyrB* genes were analysed and used to construct a phylogenetic tree. The concatenated consensus of 826 bp sequences showed 480 conserved, 345 variables, 233 parsimony-informative and 112 singleton sites (Table S5). As with individual gene phylogenies, most of the nodule DNA isolates did not show alignment with reference type strains. Due to absence of *B. vignae* 7-2^T^ in the phylogenetic tree, nodule DNA isolates TUTCSA3B, TUTCSA3A, and TUTCSA4B grouped together without any reference type strains as in the *atpD* phylogeny, but with *B. vignae* 7-2^T^ in the *glnII* phylogeny. However, TUTCT3A, TUTCS4B, and TUTCT4.I in Group III showed some closeness to *B. yuanmingense* CCBAU 10071^T^ with 94.8–96.3% sequence identity. Nodule DNA isolates TUTCSA2B, TUTCG4.I, TUTCT1A, TUTCG1.I, TUTCG5C, TUTCT2A, TUTCSA4A, TUTCSA4C and TUTCT4A in Group IV were proximally related to *B. elkanii* USDA76^T^ with 94.2–96.9% sequence identity (Fig. 2).

**Fig. 3:**
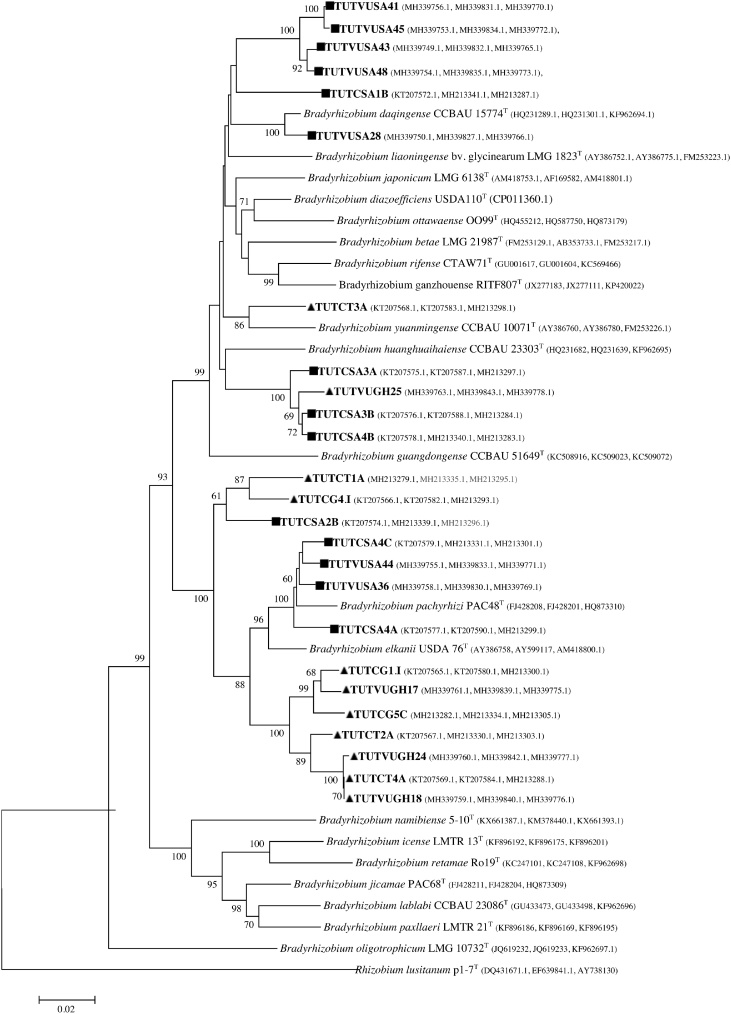
A clear view of South African (■) and Ghanaian (▲) cowpea root nodule isolates’ phylogenetic relationships based on concatenate gene (*atpD* + *glnII* + *gyrB*) sequences of present and previous (Mohammed et al. [32]) studies. Phylogeny was inferred using the maximum-likelihood method. The percentages of replicate trees in which the associated taxa clustered together were obtained using bootstrap test with 1000 replications.

A combined phylogenetic study previously discussed by Mohammed et al. [32] and present research on cowpea root nodule isolates collected from South Africa and Ghana showed that both countries retained very diverse and novel *Bradyrhizobium* species (Fig. 3). Except the isolates TUTCT4A (present study) and TUTVUGH18 (previously studied) from Ghana, all the isolates were not closely related (Fig. 3).

### Phylogeny of the *nif*H gene sequences of bacteria from cowpea root nodules

**Fig. 4:**
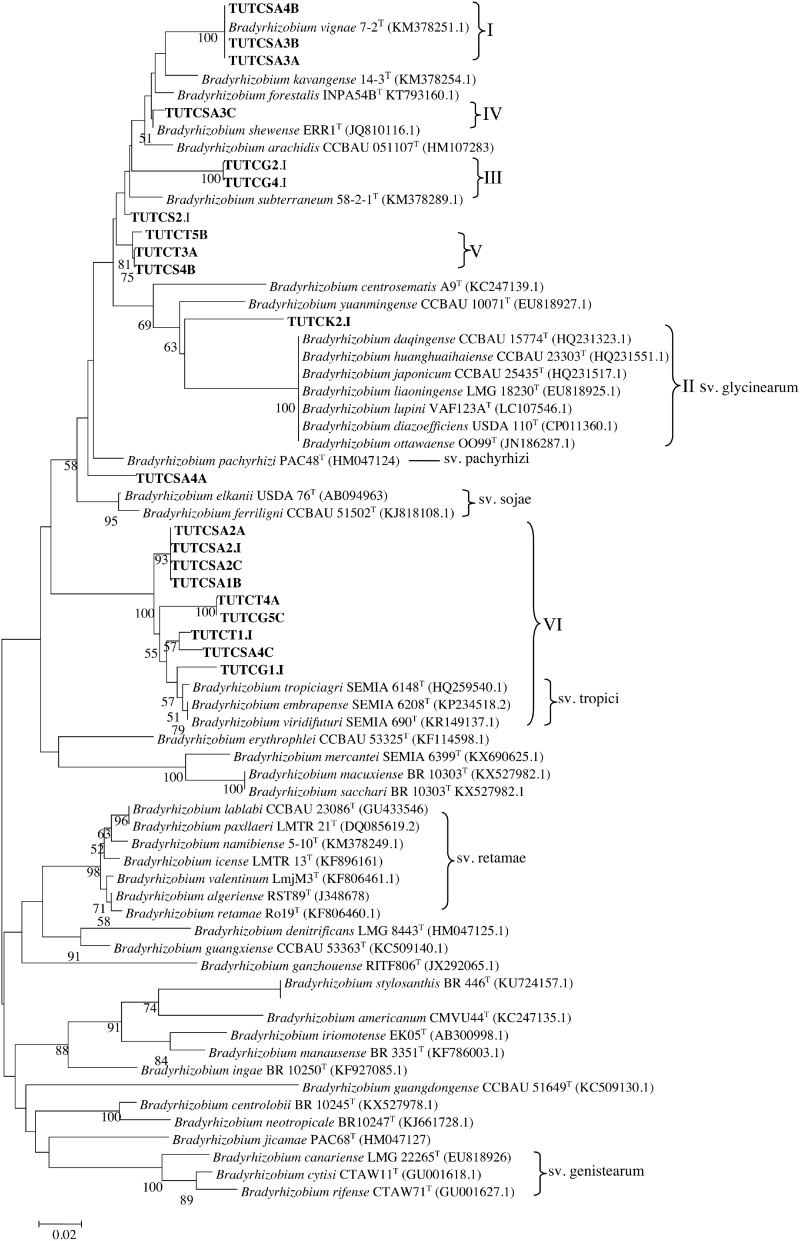
Phylogenetic relationships of *nifH* gene sequences of cowpea nodule DNA. Phylogeny was inferred using the maximum-likelihood method. The percentages of replicate trees in which the associated taxa clustered together were obtained using bootstrap test with 1000 replications.

The *nif*H gene sequences of nodule DNA isolates showed 93–100% identity with various reference type *Bradyrhizobium* species and formed six different groups (Groups I–VI) (Fig. 4). As with the housekeeping gene phylogenies, only nodule DNA isolates TUTCSA3A, TUTCSA3B and TUTCSA4B (Group I) from Morwe in South Africa closely aligned with *B. vignae* 7-2^T^ with 100% sequence identity and 95% bootstrap support. Nodule isolate TUTCK2.I which is an outgroup of *Bradyrhizobium diazoefficiens* (Group II), showed a proximal relatedness to *Bradyrhizobium yuanmingense* CCBAU10071^T^ with 93% sequence identity. Placed together in Group III without any type reference strains, nodule DNA isolates TUTCG2.I and TUTCG4.I showed 100% sequence identity and revealed proximal closeness with *B. subterraneum* 58-2-2^T^ with 95.6% sequence identity. Nodule DNA isolate TUTCSA3C in Group IV was however grouped with *B. arachidis* with 97.5% sequence identity, while those in Groups V and VI stood separately without any reference type strains.

**Fig. 5:**
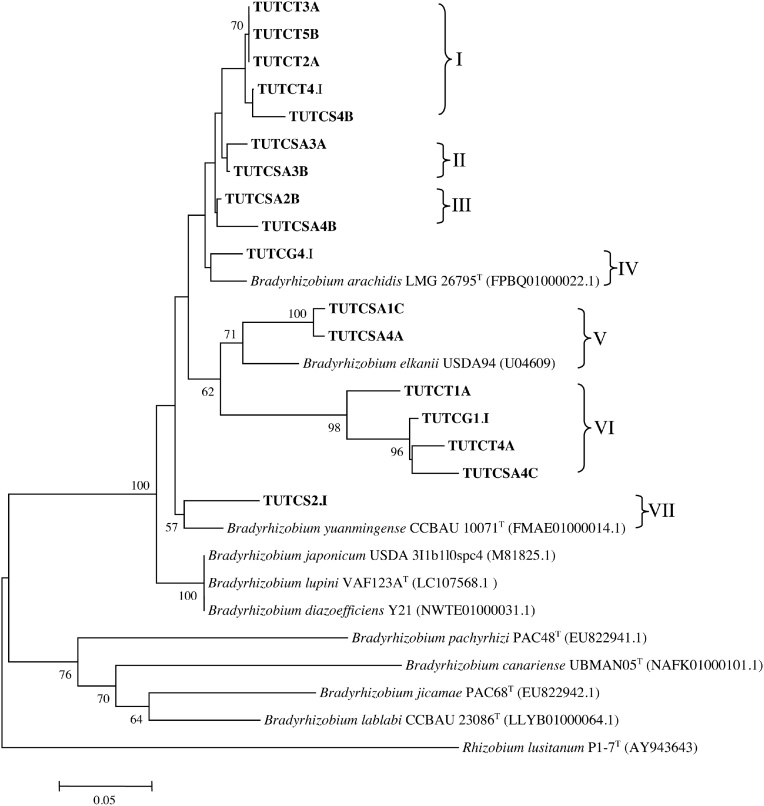
Phylogenetic relationships of *nodD* gene sequences of cowpea nodule DNA. Phylogeny was inferred using the maximum-likelihood method. The percentages of replicate trees in which the associated taxa clustered together were obtained using bootstrap test with 1000 replications.

The nodulation gene (*nodD*) phylogenetic analysis placed the nodule DNA isolates into seven (I–VII) groups without any close reference type strains. However, *Bradyrhizobium elkanii* USDA 94 was the nearest strain to nodule DNA isolates TUTCSA1C and TUTCSA4A in Group V and isolate TUTCS2.I grouped with *B. yuanmingense* CCBAU 10071^T^ in Group VII. The nodule DNA isolates in Groups I–IV and VI stood alone without any reference type strains (Fig. 5).

## Discussion

Our study revealed the distribution of microsymbionts nodulating cowpea grown in soils with different physico-chemical properties from different agro-ecosystems of Ghana and South Africa. Since there was no history of bacterial inoculation in the test sites, the micrsymbionts identified were considered to be indigenous strains with great adaptability to those regions, and thus provided unique concepts and information from West and Southern African ecosystems. In this study, bacterial occupants of root nodules from five cowpea genotypes were assessed using 16S–23S rRNA ITS-RFLP. PCR amplification of the ITS region of nodule DNA isolates produced prominent single bands of between 573 and 1298 bp, which were grouped into 28 ITS types. The results of other studies also reported polymorphic ITS bands in cowpea-nodulating isolates from African soils [9], [16], [26]. The presence of high variation in the ITS sequence region has been reported and suggested to be a useful marker for differentiating closely related *Bradyrhizobium* species [9], [22], [51], [58]. The differences in bands sizes and number of ITS types in results between this study and previous reports probably suggests variation in the conserved blocks, as well as the presence (or absence) of tRNA within the ITS region [17], [27], [29], [54], [58].

The restriction digestion of 76 nodules DNA samples of five cowpea genotypes with different seedcoat pigmentations and planted in six locations, produced 30 ITS PCR-RFLP patterns which grouped into five major clusters (Fig. S2). This is a very high number of PCR-RFLP ITS types when compared to the findings of previous studies [9], [26], [40], and this could be attributed to the number and choice of restriction enzymes used. The ITS amplicons of some of the test isolates could not be digested by some of the restriction enzymes used in this study (Table 1), thus suggesting the absence of their respective restriction sites in the ITS sequences [9], [23], [38].

Furthermore, the highest number of ITS-RFLP types were found in cowpea nodules from Morwe in South Africa, and this could suggest the presence of a more diverse cowpea-nodulating rhizobial population at the Morwe site (Table 1). This finding is consistent with that of Pule-meulenberg et al. [40] who showed that the diversity of bradyrhizobia nodulating cowpea was higher in South Africa when compared to Ghana. It is important to note that while drought (or low-water-deficit) can suppress nodulation and N_2_ fixation in legumes [21], Krasova-wade et al. [26] found higher diversity of cowpea-nodulating rhizobia in drier environments. Law et al. [28] also reported high rhizobial richness in the low rainfall areas of South Africa and Botswana. Recently, Gronemeyer et al. [16] and Ndungu et al. [37], also found greater rhizobial diversity in the semi-arid than humid areas of Namibia and Kenya, respectively. In this study, the rainfall at Morwe in South Africa site was very low when compared to the study sites in Ghana. But whether the lower rainfall at Morwe contributed to the higher diversity of cowpea-nodulating rhizobia at that site, remains to be determined.

The legume rhizosphere can enrich some microbial populations due to the effects of the legume genotype on the soil microbial ecology [15], [35], [42], [53]. The cowpea varieties used in this study appeared to have substantially affected the choice of microsymbiont for nodule formation on root hairs. This was evidenced by the differences observed among the ITS types and ITS PCR-RFLP patterns (Table 1; Fig. S2) of nodules from the different cowpea varieties planted in the same hole. For instance, nodule DNA isolates TUTCG1.I, TUTCG2.I and TUTCG4.I respectively, obtained from nodules of the Black, Red and Cream cowpea varieties differed in their ITS-RFLP types (Table 1, Fig. S2), although they had access to the same rhizosphere population of rhizobia. In the phylogenetic studies, TUTCG1.I grouped with *B. elkanii*, while TUTCG2.I and TUTCG4.I aligned with *B. japonicum* group (see Figs. S4–S6). The observed differences in rhizobial diversity could be due to the profile and concentrations of *nod*-gene inducing flavonoids and anthocyanins present in the seed and root exudates of the cowpea genotypes studied [30], [31], [36], [42], [57]. The cowpea varieties with different seedcoat pigmentations clearly selected for different root-nodule bacteria, as no particular ribotypes were selected in all the sampling sites by the same genotype. Both plant and soil factors may have therefore acted together to determine the diversity of cowpea-nodulating rhizobia. But the mechanistic details of the effect of seedcoat colour on rhizobial diversity remains to be determined.

Environmental factors, including soil physico-chemistry, can influence all aspects of legume nodulation and N_2_ fixation, and are sometimes responsible for bacterial community structure and function in soils [22], [37]. In this study, the ITS-RFLP data of the nodule DNA isolates obtained from acidic African soils in Ghana and South Africa, and the CCA analysis seem to suggest the existence of a relationship between bradyrhizobial population and the physicochemical properties of soil. For example, nodule isolates TUTCT1C, TUTCT4A, TUTCT4.I, TUTCG5B and TUTCG1C from Ghana were strongly influenced by the soil concentration of Zn, N, and K, and were thus highly diverse among the test bacterial populations (Fig. 1B and C). In contrast, nodule isolate TUTCG5C was highly influenced by soil organic matter (Fig. 1A). Ndungu et al. [37] reported recently that the occurrence and abundance of diverse cowpea rhizobial populations in Kenya was influenced by soil pH, which often affects the bioavailability of mineral nutrients in soils. The results of the study suggest that the bradyrhizobial community structure and composition were strongly controlled by soil physico-chemical properties such as pH, organic matter and macro-elements (e.g. P), as well as micro-elements (e.g. Zn). Thus, the edaphic effect on the bradyrhizobial community was indirectly driven by the agro-ecosystem.

The sequence information from 16S rRNA, nodulation and N_2_ fixation genes *nodD* and *nif*H, and as well as the housekeeping genes *atp*D, *gyrB* and *gln*II were used to assess the identity of microsymbionts in cowpea root nodules. Phylogenies constructed from sequences of single genes revealed differences in the evolutionary relationships among the nodule occupants. For example, nodule DNA isolate TUTCT1A aligned with *B. iriomotense* in the *atpD* phylogeny, but with *B. elkanii* in the *glnII* and *gyrB* phylogenies. Nodule DNA isolates TUTCSA2B and TUTCG4.I also respectively stood alone in the *atpD* and *gyrB* phylogenies, but aligned with *B. elkanii* in the *glnII* phylogeny. The observed incongruencies in single gene phylogenies among nodule DNA isolates could be attributed to differences in the evolutionary history of the genes, recombination, and/or horizontal gene transfer [9], [34]. In this study, nodule DNA isolates TUTCSA3A, TUTCSA3B and TUCSA4B consistently grouped with *B. vignae* 7-2^T^ which was originally isolated from cowpea in the Okavango region of Namibia, and could effectively nodulate *Vigna subterranea*, *Vigna unguiculata*, *Arachis hypogaea* and on *Lablab purpureus* [16]. This probably shows the greater adaptability of *B. vignae* to African regions. However most of the nodule DNA isolates did not align with any known *Bradyrhizobium* type strains. For example, nodule DNA isolates TUTCT5B, TUTCG5C, TUTCT1A, TUTCT3A, TUTCG1.I, TUTCG2.I and TUTCG4.I showed no close relationship with any known reference *Bradyrhizobium* type strains in both the 16S rRNA and concatenated phylogenies, and could therefore be novel groups within the genus *Bradyrhizobium*. Concatenated phylogeny constructed from sequence results previously reported by Mohammed et al.[32] and present results indicated that presence of diverse and novel *Bradyrhizobium* sp. nodulating cowpea in both South African and Ghanaian soils.

Nodule DNA isolates TUTCSA3A, TUTCSA3B and TUCSA4B aligned with *B. vignae* in the phylogenetic trees of 16S rRNA, housekeeping and *nif*H genes, while nodule DNA isolates TUTCT5B and TUTCSA2A closely aligned respectively with *B. kavangense* and *B. elkanii* in the 16S rRNA phylogeny, yet stood as a separate group in the *nifH* phylogeny. This could mean that these test root-nodule bacteria acquired their N_2_ fixation gene through recombination, migration or lateral gene transfer [2], [9], [25], [45], [55]. Furthermore, nodule DNA isolates TUTCG2.I, TUTCG4.I, TUTCT4A, TUTCT2A, TUTCG5C, TUTCT1.I, TUTCSA4C, and TUTCSA2.I showed very low similarities with the reference type *Bradyrhizobium* strains in all the phylogenetic trees, and could therefore represent novel microsymbiont species within the *Bradyrhizobium* group.

The establishment of a legume-microsymbiont interaction is often accelerated by bacteria-specific plant signal molecules, usually detected by the sensory NodD proteins which play an important role in nodulation specificity. In this study, the amplification of *nodD* was not successful with some of the nodule DNA isolates, and this could be due to incompatibility with the primers used. In fact, earlier reports have found that rhizobia can possess more than one copy of the *nodD* gene, each with different properties within the same strain or one *Rhizobium* species [6]. Although the amplified *nodD* region yielded single bands with the same base pair length for all the nodule DNA samples tested, the sequences differed, as they formed five different clusters in the *nodD* phylogeny. Only nodule DNA isolates TUTCS2.I; TUTCSA1C and TUTCSA4A showed proximal relation with *Bradyrhizobium yuanmingense* CCBAU 10071^T^ and *B. elkanii* USDA 94 isolated from root nodules of *Lespedeza* and *Glycine max,* respectively, with 92.6 and 91.5% sequence identity, respectively. The transfer of *nodD* gene from one bacterium to another can alter the host specificity of both the donor and recipient strains [20], [50]. Although the divergent symbiotic sequences found from the studies of *nodD* and *nifH* are interesting, we are unable to show functional differences due to the use of bacterial DNA extracted directly from root nodules. Future studies using bacterial cultures from cowpea nodules will shed light on the relationship between divergent symbiotic sequences and functional diversity, as well as differences in the symbiotic efficiency of bacteria from Ghana and South Africa, if any.

Taken together, this study has shown that high intra-species diversity existed among the bradyrhizobia nodulating cowpea in Ghana and South Africa. Nodule occupancy by diverse bradyrhizobia in cowpea genotypes sown sole or together in one hole, is controlled by the host plant. Many of the nodule bacteria from this study formed unique monophyletic groups in the 16S rRNA, *atp*D, *glnII, gyrB, nifH*, and *nodD* phylogenetic trees without any reference strains, which suggest the presence of new undefined genospecies of *Bradyrhizobium* in both Ghanaian and South African soils, as well as their extended adaptability to soils of both countries. Cowpea genotypic differences, as well as the variable soil factors of the study sites seemed to have played a critical role in the choice of microsymbiont partners.
