# Supplementary material for: Phylogeny and distribution of Bradyrhizobium symbionts nodulating cowpea (Vigna unguiculata L. Walp) and their association with the physicochemical properties of acidic African soils
Source: Syst Appl Microbiol. 2019 May;42(3):403–14. doi: 10.1016/j.syapm.2019.02.004 (PMC6542415; doi:10.1016/j.syapm.2019.02.004)
Supplement: Supplementary file 1 [file mmc1.docx]

Table S1: Soil Physical and Chemical properties of sites in Ghana prior to setting up of Experiments

| Soil parameter | Texture | pH | Org C | Total N | Org matter | Ca | Mg | K | Na | CEC | P | Zn | Cu | Mn | Fe |
| --- | --- | --- | --- | --- | --- | --- | --- | --- | --- | --- | --- | --- | --- | --- | --- |
|  | | | % | mg.kg^-1^ | | | | | | | | | | | |
| Kpalisogu | Sandy loam | 4.7 | 0.51 | 0.05 | 0.88 | 320 | 96 | 59 | 9 | 484 | 8 | 10.8 | 3.9 | 32 | 1.50 |
| Gbalahi | Sandy loam | 5.1 | 0.63 | 0.06 | 1.09 | 640 | 224 | 47 | 7 | 918 | 7 | 20.8 | 3.4 | 25 | 2.35 |
| Manga | Sandy loam | 4.7 | 0.48 | 0.05 | 0.83 | 214 | 32 | 98 | 12 | 355 | 23 | 12.8 | 4.6 | 18 | 1.40 |
| Googo | Sandy loam | 4.6 | 0.9 | 0.08 | 1.38 | 534 | 168 | 140 | 16 | 851 | 15 | 9.9 | 8.2 | 70 | 1.95 |
| Savelugu | Sandy loam | 4.9 | 0.39 | 0.04 | 0.67 | 320 | 64 | 55 | 7 | 445 | 8 | 1.8 | 6.3 | 30 | 0.95 |
| Morwe | Sandy loam | 5.5 | 0.57 | 0.02 | 0.96 | 164 | 38 | 128 | 11 | 341 | 10 | 1.3 | 0.5 | 59 | ND |

Table S2: List of primer sets and the respective temperature profiles used in the PCR

| **Target genes** | **PCR temperature profile** | **Primer sequences** | **Reference** |
| --- | --- | --- | --- |
| IGS (16S-23S rDNA) | 3’95 ºC, 35x(1’94 ºC, 1’55 ºC, 1.45’72 ºC), 3’72 ºC | F: 5ʹTGCGGCTGGATCACCTCCTT3ʹ  R: 5ʹCCGGGTTTCCCCATTCGG3ʹ | Appunu *et* *al*., 2009 |
| 16SrRNA | 5 min 95 ̊C, 35 X (1 min 95 ̊C, 1 min 55 ̊C, 1 min 72 ̊C), 10 min 72 | F-5’AGAGTTTGATCCTGGCTCAG3’  R-5’CTTAAGGAGGTGATCCAGCC3’ | Weisburg et al. 1991 |
| *nifH* | 5’94 ºC, 20x(0:30’94 ºC, 0:30’65 ºC, 0.5 per cycle, 1:30’72 ºC), 25x(0:30’94 ºC, 0:30’55 ºC, 1:30’72 ºC), 10’72 ºC | F:5ʹTACGGNAARGGSGGNATCGGCAA3ʹ  R:5ʹAGCATGTCYTCSAGYTCNTCCA3ʹ | Nzoue *et* *al*., 2009 |
| *atpD* | 2’95 ºC, 35x(0.45’95 ºC, 0:30’65 ºC, 1:30’72 ºC), 10`72 ºC | F: 5'TCTGGTCCGYGGCCAGGAAG3'  R: 5'CGACACTTCCGARCCSGCCTG3' | Stepkowski *et* *al*.,2011 |
| *glnII* | 2’95 ºC, 35x(0.45’95 ºC, 0:30’65 ºC, 1:30’72 ºC), 10`72 ºC | F: 5'AAGCTCGAGTACATCTGGCTCGACGG3'  R: 5'SGAGCCGTTCCAGTCGGTGTCG3' | Stepkowski *et* *al*.,2011 |
| *gyrB* | 10 min 95 ̊C, 35 X (30 s 94 ̊C, 30 s 58 ̊C, 1 min 72 ̊C), 10 min 72 ̊ C | 343f-TTCGACCAGAAYTCCTAYAAGG  1043r-AGCTTGTCCTTSGTCTGCG | Marek-Kozaczuk et al. (2013) |
| *nodD* | 0.30’95 ºC, 40x(0.30’96 ºC, 1’53 ºC, 0:30’72 ºC), 05`72 ºC | F-5’GAT YGT CAT GAA ATC KGA GAG3’  R-5’TCG ATA GAA NAC ATC CAC ACG AT3’ | Sterner and Parker 1999 |

1. Appunu C, Sasirekha N, Prabavathy RV, Nair S. 2009. A significant proportion of indigenous rhizobia from India associated with soybean (*Glycine max* L.) distinctly belong to *Bradyrhizobium*and *Ensifer* genera. Biol Fertil Soils 46:57-63.
2. Weisburg WG, Barns SM, Pelletier DA, Lane DJ. 16S ribossomal DNA amplification for phylogenetic study. J Bacteriol. 1991;173: 697-703.
3. Marek-Kozaczuk M, Leszcz A, Wielbo J, Wdowiak-Wróbel S, Skorupska A. 2013. *Rhizobiumpisi*sv. *trifolii* K3. 22 harboring nod genes of the *Rhizobium leguminosarum* sv. *trifolii* cluster. Syst Appl Microbiol 36(4):252-258.
4. Nzoué A, Miché L, Klonowska A, Laguerre G, de Lajudie P, Moulin L. Multilocus sequence analysis of bradyrhizobia isolated from Aeschynomene species in Senegal. Syst Appl Microbiol. 2009;32**:**400-412.
5. Stępkowski T, Żak M, Moulin L, Króliczak J, Golińska B, Narożna D, ... Mądrzak CJ. *Bradyrhizobium* *canariense* and *Bradyrhizobium* *japonicum* are the two dominant *Rhizobium* species in root nodules of lupin and serradella plants growing in Europe. Syst Appl Microbiol. 2011;34**:** 368-375.
6. Sterner JP, Parker MA. Diversity and relationships of bradyrhizobia from Amphicarpaea bracteata based on partial nod and ribosomal sequences. Syst Appl Microbiol. 1999;2(3):387-392.

**Table S3.** GenBank accession number of the gene sequences used in this study for cowpea nodulating rhizobial isolates

| **Isolates** | **16S rRNA** | ***atpD*** | ***glnII*** | ***GyrB*** | ***nifH*** | ***nodD*** |
| --- | --- | --- | --- | --- | --- | --- |
| **TUTCT2A** | MH213327 | KT207567 | MH213330 | MH213303 | KT207595 | KY355102 |
| **TUTCSA4C** | MH213326 | KT207579 | MH213331 | MH213301 | KT207607 |  |
| **TUTCT1.I** | MH213324 |  | MH213332 | MH213291 | KT207598 |  |
| **TUTCSA2.I** | MH213323 |  | KT207591 | MH213290 | KT207609 |  |
| **TUTCSA2A** | MH213322 |  | KT207585 | MH213286 | MH208457 |  |
| **TUTCT4A** | MH213320 | KT207569 | KT207584 | MH213288 | KT207597 | KY355104 |
| **TUTCSA4A** | MH213316 | KT207577 | KT207590 | MH213299 | KT207605 | KY355111 |
| **TUTCSA2C** | MH213312 |  | KT207586 | MH213285 | KT207601 |  |
| **TUTCSA1C** | MH213311 | KT207573 | MH213333 |  |  | KY355107 |
| **TUTCG1.I** | MH213328 | KT207565 | KT207580 | MH213300 | MH208458 | KY355099 |
| **TUTCG5C** | MH213308 | MH213282 | MH213334 | MH213305 | MH208459 |  |
| **TUTCT1A** | MH213309 | MH213279 | MH213335 | MH213295 |  | KY355101 |
| **TUTCT5B** | MH213315 | KT207570 | MH213336 | MH213289 | MH208460 | KY355105 |
| **TUTCG2.I** | MH213325 |  | KT207581 | MH213292 | KT207593 |  |
| **TUTCG4.I** | MH213329 | KT207566 | KT207582 | MH213293 | KT207594 | KY355100 |
| **TUTCT3A** | MH213319 | KT207568 | KT207583 | MH213298 | KT207596 | KY355103 |
| **TUTCT4.I** | MH213310 | MH213280 | MH213337 | MH213294 |  | KY355106 |
| **TUTCS4B** | MH213313 | MH213281 | MH213338 | MH213302 | MH208461 | MH213306 |
| **TUTCSA2B** | MH213321 | KT207574 | MH213339 | MH213296 |  | KY355108 |
| **TUTCSA4B** | MH213314 | KT207578 | MH213340 | MH213283 | KT207606 | KY355112 |
| **TUTCSA3B** | MH213317 | KT207576 | KT207588 | MH213284 | KT207603 | KY355110 |
| **TUTCSA3A** | MH213318 | KT207575 | KT207587 | MH213297 | KT207602 | KY355109 |
| **TUTCK2.I** |  | KT207571 |  |  | MH208462 |  |
| **TUTCSA1B** |  | KT207572 | MH213341 | MH213287 | KT207600 |  |
| **TUTCS2.I** |  |  | MH213342 | MH213304 | KT207612 | MH213307 |
| **TUTCSA3C** |  |  | KT207589 |  | KT207604 |  |

**Table S4**: constrained and unconstrained values of different axis of CCA ordination plots

| ***Bradyrhizobium-*soil physical properties** | | | | | | | |
| --- | --- | --- | --- | --- | --- | --- | --- |
|  | **Inertia** | **proportion** | **Importance of components** | **CCA1** | **CCA2** | **CCA3** | **CCA4** |
| **Total** | 0.147 | 1.000 | **Eigenvalue** | 0.060 | 0.020 | 0.012 | 0.005 |
| **Constrained** | 0.097 | 0.665 | **Proportion Explained** | 0.409 | 0.139 | 0.083 | 0.033 |
| **Unconstrained** | 0.049 | 0.334 | **Cumulative Proportion** | 0.409 | 0.549 | 0.632 | 0.665 |
| ***Bradyrhizobium* –soil macro-elements** | | | | | | | |
| **Total** | 0.147 | 1.000 | **Eigenvalue** | 1.000 | 0.232 | 0.015 | 0.005 |
| **Constrained** | 0.147 | 1.000 | **Proportion Explained** | 0.684 | 0.158 | 0.100 | 0.036 |
| **Unconstrained** | 0.000 | 0.000 | **Cumulative Proportion** | 0.684 | 0.842 | 0.943 | 0.979 |
| ***Bradyrhizobium* –soil micro-elements** | | | | | | | |
| **Total** | 0.147 | 1.000 | **Eigenvalue** | 0.991 | 0.228 | 0.013 | 0.003 |
| **Constrained** | 0.138 | 0.944 | **Proportion Explained** | 0.674 | 0.155 | 0.092 | 0.023 |
| **Unconstrained** | 0.008 | 0.055 | **Cumulative Proportion** | 0.674 | 0.829 | 0.921 | 0.944 |

**Table S5:** Information of nucleotide sequences participated in phylogenetic analysis

| **Locus** | **No. of strains used for tree construction** | **Nucleotide sequence information** | | | | **Total*** | **Frequency**  **T/C/A/G (%)** |
| --- | --- | --- | --- | --- | --- | --- | --- |
|  |  | **Cnoserved (C)** | **Variables (V)** | **Parsimony informative (Pi)** | **Singleton (S)** |  |  |
| 16S rRNA | 65 | 486 (60.4) | 313 (38.9) | 83 (10.3) | 230 (28.6) | 805 | 20.1/22.8/25.8/31.3 |
| *gln*II | 75 | 130 (60.0) | 87 (40.0) | 74 (34.1) | 13 (6.0) | 217 | 18.2/30.0/18.3/33.5 |
| *gyr*B | 66 | 116 (46.2) | 134 (53.4) | 95 (37.8) | 39 (15.5) | 251 | 17.4/33.4/19.4/29.8 |
| *atpD* | 62 | 223 (62.1) | 136 (37.9) | 89 (24.8) | 47 (13.1) | 359 | 20.6/31.2/15.9/32.3 |
| Concatenated  (*atpD*+*glnII*+ *gyr*B) | 49 | 475 (57.4) | 351 (42.4) | 244 (29.5) | 107 (12.9) | 827 | 19.0/31.5/17.6/31.9 |
| *nif*H | 69 | 114 (57.0) | 85 (42.5) | 77 (38.5) | 8(4.0) | 200 | 20.2/31.9/21.6/26.3 |
| *nodD* | 28 | 95 (45.0) | 116 (54.9) | 85 (40.3) | 31 (14.7) | 211 | 18.6/27.4/20.9/33.1 |

**Fig. S1:** Sampling locations of Ghana and South Africa in African continent


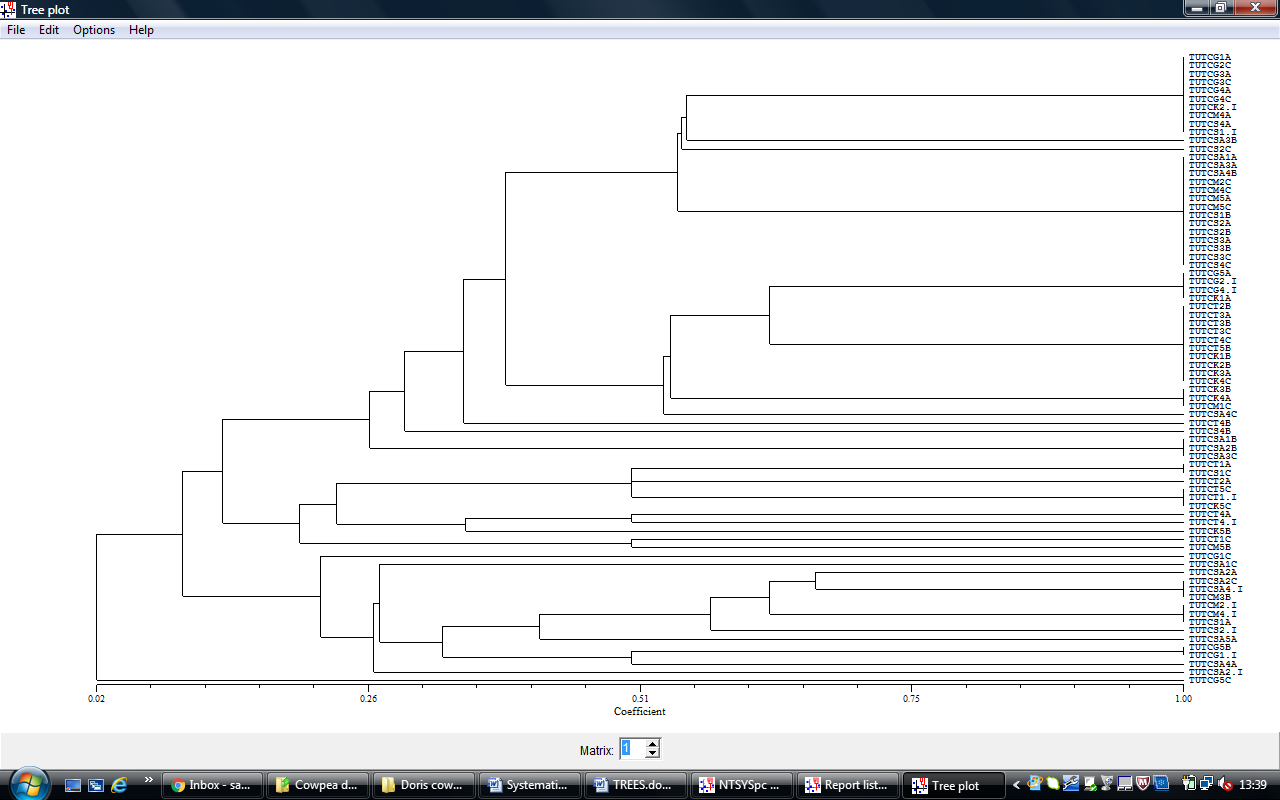


V

IV

III

II

I

**Fig. S2:** A dendrogram generated from restriction endonucleases digested ITS (16S-23S rRNA) bradyrhizobial

genomic region (ITS-RFLP) to show the similarities among the ITS-RFLP products.

**Fig S3.** Phylogenetic relationships of *16S rRNA* gene sequences of cowpea nodule DNA. Phylogeny was inferred using the maximum-likelihood method. The percentages of replicate trees in which the associated taxa clustered together were obtained using bootstrap test with 1000 replications.

together were obtained using bootstrap test with 1000 replications.

*Bradyrhizobium pachyrhizi* PAC48^T^ (NR 043037)

*Bradyrhizobium elkanii* ^T^ (U35000)

**TUTCT2A**

**TUTCSA4C**

**TUTCT1**.I

**TUTCSA2**.I

**TUTCSA2A**

**TUTCT4A**

**TUTCSA4A**

**TUTCSA2C**

**TUTCSA1C**

*Bradyrhizobium* *tropiciagri* SEMIA 6148^T^ (NR 145862)

**TUTCG1**.I

*Bradyrhizobium* *ferriligni*^T^ (NR 135878)

**TUTCG5C**

**TUTCT1A**

*Bradyrhizobium* *canariense* NBRC 103049^T^ (NR 114199)

*Bradyrhizobium* *lupini* USDA 3051^T^ (NR 134836)

*Bradyrhizobium* *lablabi* CCBAU 23086^T^ (NR 117513)

*Bradyrhizobium* *jicamae* PAC68^T^ (AY624134)

*Bradyrhizobium* *namibiense* 5-10^T^ (KX661401.2)

*Bradyrhizobium* *embrapense* SEMIA 6208^T^ (NR 145861)

*Bradyrhizobium* *erythrophlei*^T^ (NR 135877)

*Bradyrhizobium* *icense* LMTR 13^T^ (NR 133707)

*Bradyrhizobium* *retamae* Ro19^T^ (NR 118548)

*Bradyrhizobium* *viridifuturi* SEMIA 690^T^ (NR 145860)

*Bradyrhizobium* *paxallaeri* LMTR 21^T^ (AY923031)

*Bradyrhizobium* *valentinum* LmjM3^T^ (NR 125638)

*Bradyrhizobium* *mercantei* SEMIA 6399^T^ (FJ025102.1)

*Bradyrhizobium* *neotropicale* BR 10247^T^ (KF927051)

*Bradyrhizobium* *liaoningense* 2281^T^ (USDA 3622) (AF208513)

*Bradyrhizobium* *japonicum* LMG 6138^T^ (X66024)

Bradyrhizobium americanum CMVU44 (KU991833)

*Bradyrhizobium* *daqingense* CCBAU 15774^T^ (NR 118648)

*Bradyrhizobium* *ottawaense* OO99^T^ (NR 133988)

**TUTCT5B**

**TUTCG2**.I

**TUTCG4**.I

**TUTCT3A**

**TUTCT4**.I

**TUTCS4B**

**TUTCSA2B**

*Bradyrhizobium* *kavangense* 14-3^T^ (NR 145925)

*Bradyrhizobium* *subterraneum* 58 2-1^T^ (NR 137331)

*Bradyrhizobium* *yuanmingense* LMG 21827^T^ (AF193818)

*Bradyrhizobium* *oligotrophicum* S58^T^ (NR 118384)

*Bradyrhizobium* *betae* NBRC 103048^T^ (NR 114198)

*Bradyrhizobium* *diazoefficiens* USDA 110^T^ (BA000040)

*Bradyrhizobium* *centrosemae* A9^T^ (KC247115)

*Bradyrhizobium* *denitrificans* LMG 8443^T^ (NR 118982)

*Bradyrhizobium* *guangxiense* CCBAU 53363^T^ (NR 145894)

*Bradyrhizobium* *huanghuaihaiense* CCBAU 23303^T^ (NR 117945)

*Bradyrhizobium* *ingae* BR 10250^T^ (KF927043)

*Bradyrhizobium* *iriomotense* NBRC 102520^T^ (NR 114138)

*Bradyrhizobium* *stylosanthis* BR 446^T^ (KU724142)

*Bradyrhizobium* *cytisi* CTAW11^T^ (NR 116360)

*Bradyrhizobium* *rifense* CTAW71^T^ (NR 116361)

*Bradyrhizobium* *ganzhouense* RITF806^T^ (NR 133706)

*Bradyrhizobium* *guangdongense* CCBAU 51649^T^ (NR 145893)

*Bradyrhizobium* *manausense* BR 3351^T^ (NR 133986)

**TUTCSA4B**

*Bradyrhizobium* *vignae* 7-2^T^ (KP899563)

**TUTCSA3B**

**TUTCSA3A**

*Rhizobium* *lusitanum* P1-7^T^ (AY738130)

73

68

69

57

83

62

73

0.005

I

II

III

IV

**Fig. S4:** Phylogenetic relationships of *atpD* gene sequences of cowpea nodule DNA. Phylogeny was inferred using the neighbour-joining method. The percentages of replicate trees in which the associated taxa clustered together were obtained using bootstrap test with 1000 replications.

*Bradyrhizobium elkanii* USDA 76^T^ (AY386758)

*Bradyrhizobium pachyrhizi* PAC48^T^ (FJ428208)

*Bradyrhizobium macuxiense* BR 10303^T^ (LNCU01000024)

*Bradyrhizobium embrapense* SEMIA 6208^T^ (HQ634875)

**TUTCSA1C**

**TUTCSA4A**

*Bradyrhizobium mercantei* SEMIA 6399^T^ (NZ MKFI01000006)

**TUTCG1**.I

**TUTCT2A**

**TUTCT4A**

**TUTCSA4C**

*Bradyrhizobium* *brasilense* UFLA03-321^T^ (KF452730.1)

**TUTCG5C**

*Bradyrhizobium* *tropiciagri* SEMIA 6148^T^ (FJ390968)

*Bradyrhizobium* *iriomotense*^T^ (AB300994.1)

*Bradyrhizobium* *ingae* BR 10250^T^ (KY753593.1)

*Bradyrhizobium* *yuanmingense* CCBAU 10071^T^ (AY386760)

*Bradyrhizobium* *forestalis* INPA54B^T^ (KF452722.1)

*Bradyrhizobium* *subterraneum* 58 2-1^T^ (KX661391.1)

**TUTCT1A**

**TUTCG4**.I

**TUTCT3A**

**TUTCT5B**

**TUTCT4**.I

*Bradyrhizobium* *ganzhouense* RITF807^T^ (JX277183)

*Bradyrhizobium* *guangdongense* CCBAU 51649^T^ (KC508916)

*Bradyrhizobium* *diazoefficiens* USDA 110^T^ (CP011360.1)

*Bradyrhizobium* *japonicum* LMG 6138^T^ (AM418753.1)

*Bradyrhizobium* *betae* LMG 21987^T^ (FM253129.1)

*Bradyrhizobium* *ottawaense* OO99^T^ (HQ455212)

*Bradyrhizobium* *shewense* ERR11^T^ (NZ_FMAI01000019)

*Bradyrhizobium* *daqingense* CCBAU 15774^T^ (HQ231289)

*Bradyrhizobium* *americanum* CMVU44^T^ (KC247125.1)

*Bradyrhizobium* *huanghuaihaiense* CCBAU 23303^T^ (HQ231682)

*Bradyrhizobium* *rifense* CTAW71^T^ (GU001617)

*Bradyrhizobium* *guangxiense* CCBAU 53363^T^ (KC508926)

**TUTCSA3A**

**TUTCSA3B**

**TUTCSA4B**

*Bradyrhizobium* *liaoningense* bv. *glycinearum* LMG 18230^T^ (AY386752)

*Bradyrhizobium* *cytisi* CTAW11^T^ (GU001613)

*Bradyrhizobium* *canariense* LMG 22265^T^ (FM253135)

*Bradyrhizobium* *lupini* USDA 3051^T^ (KU738808.1)

*Bradyrhizobium* *arachidis* CCBAU 45332^T^ (JQ011347.1)

*Bradyrhizobium* *sacchari* BR10280^T^ (KX065107.1)

**TUTCK2**.I

**TUTCSA1B**

*Bradyrhizobium* *kavangense* 14-3^T^ (KY753592.1)

*Bradyrhizobium* *oligotrophicum* LMG 10732^T^ (JQ619232)

*Bradyrhizobium* *denitrificans* LMG 8443^T^ (FM253153.1)

**TUTCSA2B**

*Bradyrhizobium* *centrosematis* A9^T^ (KC247129.1)

*Bradyrhizobium* *lablabi* CCBAU 23086^T^ (GU433473)

*Bradyrhizobium* *icense* LMTR 13^T^ (KF896192)

*Bradyrhizobium* *retamae* Ro19^T^ (KC247101)

*Bradyrhizobium* *algeriense* RST89^T^ (KF956544.1)

*Bradyrhizobium* *valentinum* LmjM3^T^ (JX518561)

*Bradyrhizobium* *jicamae* PAC68^T^ (FJ428211)

*Bradyrhizobium* *paxllaeri* LMTR 21^T^ (KF896186)

*Bradyrhizobium* *namibiense* 5-10^T^ (KX661387.1)

**TUTCS4B**

*Rhizobium* *lusitanum* p1-7^T^

92

100

70

100

100

88

97

94

87

99

52

71

89

68

60

90

64

68

61

0.02

**Fig. S5:** Phylogenetic relationships of *glnII* gene sequences of cowpea nodule DNA. Phylogeny was inferred using the neighbour-joining method. The percentages of replicate trees in which the associated taxa clustered together were obtained using bootstrap test with 1000 replications.

*Bradyrhizobium diazoefficiens* USDA 110^T^ ( CP011360.1)

*Bradyrhizobium* *ottawaense* OO99^T^ (HQ587750)

*Bradyrhizobium* *shewense* ERR11^T^ (JQ809893.1)

*Bradyrhizobium* *betae* LMG 21987^T^ (AB353733.1)

*Bradyrhizobium* *japonicum* LMG 6138^T^ (AF169582)

*Bradyrhizobium* *americanum* CMVU44^T^ (KX012942)

*Bradyrhizobium* *daqingense* CCBAU 15774^T^ (HQ231301)

*Bradyrhizobium* *ganzhouense* RITF807^T^ (JX277111)

*Bradyrhizobium* *stylosanthis* BR 446^T^ (KU724148.1)

*Bradyrhizobium* *cajani* AMBPC1010^T^ (KY349442.1)

*Bradyrhizobium* *huanghuaihaiense* CCBAU 23303^T^ (HQ231639)

**TUTCSA1B**

*Bradyrhizobium* *canariense* bv. *genistearum* BTA-1^T^ (AY386765.1)

*Bradyrhizobium* *lupini* USDA 3051^T^ (KM114862)

*Bradyrhizobium* *cytisi* CTAW11^T^ (GU001594)

*Bradyrhizobium* *rifense* CTAW71^T^ (GU001604)

*Bradyrhizobium* *guangdongense* CCBAU 51649^T^ (KC509023)

*Bradyrhizobium* *centrolobii* BR 10245^T^ (KX527991.1)

*Bradyrhizobium* *neotropicale* BR 10247^T^ (KJ661700.1)

**TUTCSA4B**

*Bradyrhizobium* *vignae* 7-2^T^ (KM378443)

**TUTCSA3B**

**TUTCSA3A**

*Bradyrhizobium* *centrosematis* A9^T^ (KX012940.1)

*Bradyrhizobium* *guangxiense* CCBAU 53363^T^ (KC509033)

**TUTCSA3C**

*Bradyrhizobium* *arachidis* CCBAU 051107^T^ (HM107251)

*Bradyrhizobium* *liaoningense* bv. *glycinearum* LMG 18230^T^ (AY386775)

**TUTCG2**.I

**TUTCS4B**

**TUTCT4**.**I**

**TUTCT3A**

**TUTCT5B**

*Bradyrhizobium* *kavangense* 14-3^T^ (KM378446)

*Bradyrhizobium* *ingae* BR 10250^T^ (KF927067)

*Bradyrhizobium* *iriomotense* (AB300995.1)

**TUTCS2**.I

*Bradyrhizobium* *subterraneum* 60 2-1 ^T^ (KM378485.1)

*Bradyrhizobium* *yuanmingense* CCBAU 10071^T^ (AY386780)

*Bradyrhizobium* *manausense* BR 3351^T^ (KF785986)

*Bradyrhizobium* *erythrophlei* CCBAU 53325^T^ (KF114693)

**TUTCT1A**

**TUTCSA2B**

**TUTCG4.I**

*Bradyrhizobium* *elkanii* USDA 76^T^ (AY599117)

*Bradyrhizobium* *macuxiense* BR 10303^T^ (KX527995.1)

*Bradyrhizobium* *sacchari* BR 10303^T^ (KX527995.1)

**TUTCSA2A**

**TUTCSA2**.I

**TUTCSA2C**

**TUTCT1.I**

**TUTCT4A**

**TUTCT2A**

**TUTCG1.I**

**TUTCG5C**

*Bradyrhizobium* *viridifuturi* SEMIA 690^T^ (KR149131)

*Bradyrhizobium* *embrapense* SEMIA 6208^T^ (GQ160500)

*Bradyrhizobium* *tropiciagri* SEMIA 6148^T^ (FJ391048)

*Bradyrhizobium* *mercantei* SEMIA 6399^T^ (KX690621.1)

**TUTCSA4A**

**TUTCSA1C**

*Bradyrhizobium* *ferriligni* CCBAU 51502^T^ (KJ818099)

**TUTCSA4C**

*Bradyrhizobium* *pachyrhizi* PAC48^T^ (FJ428201)

*Bradyrhizobium* *namibiense* 5-10^T^ (KM378440.1)

*Bradyrhizobium* *icense* LMTR 13^T^ (KF896175)

*Bradyrhizobium* *retamae* Ro19^T^ (KC247108)

*Bradyrhizobium* *algeriense* RST89^T^ (FJ264924.1)

*Bradyrhizobium* *valentinum* LmjM3^T^ (JX518575)

*Bradyrhizobium* *jicamae* PAC68^T^ (FJ428204)

*Bradyrhizobium* *lablabi* CCBAU 23086^T^ (GU433498)

*Bradyrhizobium* *paxllaeri* LMTR 21^T^ (KF896169)

*Bradyrhizobium* *denitrificans* LMG 8443^T^ (HM047121)

*Bradyrhizobium* *oligotrophicum* LMG 10732^T^ (JQ619233)

*Rhizobium* *lusitanum* P1-7^T^ (EF639841.1)

83

87

61

77

100

85

72

71

95

55

76

87

71

70

50

77

91

93

100

67

58

59

77

89

69

55

81

74

81

64

87

79

50

78

0.02

**Fig. S6:** Phylogenetic relationships of *gyrB* gene sequences of cowpea nodule DNA. Phylogeny was inferred using the neighbour-joining method. The percentages of replicate trees in which the associated taxa clustered together were obtained using bootstrap test with 1000 replications.

*Bradyrhizobium* *arachidis* CCBAU 45332^T^ (JX437674.1)

*Bradyrhizobium* *stylosanthis* BR 446^T^ (KU724151.1)

**TUTCSA1B**

**TUTCSA2B**

*Bradyrhizobium* *japonicum* LMG 6138^T^ (AM418801.1)

*Bradyrhizobium* *liaoningense* LMG 18230^T^ (FM253223.1)

*Bradyrhizobium* *kavangense* 14-3^T^(KX661397.1)

*Bradyrhizobium* *centrolobii* BR 10245^T^ (KX528004.1)

*Bradyrhizobium* *ingae* BR 10250^T^ (KF927079)

*Bradyrhizobium* *neotropicale* BR 10247^T^ (KJ661707.1)

*Bradyrhizobium* *guangdongense* CCBAU 51649^T^ (KC509072)

*Bradyrhizobium* *manausense* BR 3351^T^ (KF786000.1)

*Bradyrhizobium* *diazoefficiens* USDA 110^T^ (CP011360.1)

*Bradyrhizobium* *ottawaense* OO99^T^ (HQ873179)

*Bradyrhizobium* *huanghuaihaiense* CCBAU 23303^T^ (KF962695)

*Bradyrhizobium* *canariense* LMG 22265^T^ (FM253220.1)

*Bradyrhizobium* *betae* LMG 21987^T^ (FM253217.1)

*Bradyrhizobium* *ganzhouense*^T^ (KP420022)

*Bradyrhizobium* *rifense* CTAW71^T^ (KC569466)

*Bradyrhizobium* *daqingense* CCBAU 15774^T^ (KF962694)

*Bradyrhizobium* *guangxiense* CCBAU 53363^T^ (KC509082)

**TUTCG2.I**

**TUTCG4.I**

**TUTCS2**.I

*Bradyrhizobium* *yuanmingense* LMG 21827^T^ (FM253226.1)

**TUTCT3A**

**TUTCS4B**

**TUTCT5B**

**TUTCT4.I**

*Bradyrhizobium* *forestalis* INPA54B^T^ (KF452831.1)

*Bradyrhizobium* *denitrificans* LMG 8443^T^ (FM253239.1)

*Bradyrhizobium* *subterraneum* 58 2-1^T^ (KX661396.1)

**TUTCSA3A**

**TUTCSA3B**

**TUTCSA4B**

*Bradyrhizobium* *vignae* 7-2^T^(KX683216.1)

**TUTCT2A**

*Bradyrhizobium* *macuxiense* BR 10303^T^(KX528008.1)

*Bradyrhizobium* *sacchari* BR 10303^T^ (KX528008.1)

**TUTCSA2C**

**TUTCSA2A**

**TUTCSA2.I**

**TUTCT1.I**

**TUTCT1A**

**TUTCSA4C**

*Bradyrhizobium* *brasilense* UFLA03-321^T^(KF452827.1)

*Bradyrhizobium* *elkanii* LMG 6134^T^(AM418800.1)

*Bradyrhizobium* *pachyrhizi* PAC 48^T^(HQ873310)

**TUTCSA4A**

**TUTCG1**.I

**TUTCG5C**

**TUTCT4A**

*Bradyrhizobium* *mercantei* SEMIA 6399^T^(KX690623.1)

*Bradyrhizobium* *ferriligni* CCBAU 51502^T^(KJ818102)

*Bradyrhizobium* *embrapense* SEMIA 6208^T^ (HQ634891)

*Bradyrhizobium* *tropiciagri* SEMIA 6148^T^ (HQ634890)

*Bradyrhizobium* *viridifuturi* SEMIA 690^T^(KR149134)

*Bradyrhizobium* *erythrophlei* CCBAU 53325^T^ (KF114717)

*Bradyrhizobium* *namibiense* 5-10^T^(KX661393.1)

*Bradyrhizobium* *icense* LMTR 13^T^(KF896201)

*Bradyrhizobium* *retamae* Ro19^T^ (KF962698)

*Bradyrhizobium* *jicamae* PAC 68^T^(HQ873309)

*Bradyrhizobium* *lablabi* CCBAU 23086^T^(KF962696)

*Bradyrhizobium* *paxllaeri* LMTR 21^T^(KF896195)

*Bradyrhizobium* *oligotrophicum* LMG 10732^T^(KF962697.1)

*Rhizobium* *lusitanum* P1-7^T^(AY738130)

100

75

98

73

100

81

56

92

72

98

82

78

87

64

70

67

76

73

55

73

55

63

0.05
